# The deuterated pyrazoloquinolinone targeting α6 subunit-containing GABA_A_ receptor as novel candidate for inhibition of trigeminovascular system activation: implication for migraine therapy

**DOI:** 10.3389/fphar.2024.1451634

**Published:** 2024-08-26

**Authors:** Pi-Chuan Fan, Lih-Chu Chiou, Tzu-Hsuan Lai, Dishary Sharmin, James Cook, Ming Tatt Lee

**Affiliations:** ^1^ Department of Pediatrics, College of Medicine, National Taiwan University, Taipei, Taiwan; ^2^ Department of Pediatrics, National Taiwan University Hospital, Taipei, Taiwan; ^3^ Clinical Center for Neuroscience and Behavior, National Taiwan University Hospital, Taipei, Taiwan; ^4^ Department of Pharmacology, College of Medicine, National Taiwan University, Taipei, Taiwan; ^5^ Graduate Institute of Brain and Mind Sciences, College of Medicine, National Taiwan University, Taipei, Taiwan; ^6^ Graduate Institute of Acupuncture Sciences, China Medical University, Taichung, Taiwan; ^7^ Department of Chemistry and Biochemistry, University of Wisconsin-Milwaukee, Milwaukee, United States; ^8^ Faculty of Pharmaceutical Sciences, UCSI University, Kuala Lumpur, Malaysia; ^9^ UCSI Wellbeing Research Centre, UCSI University, Kuala Lumpur, Malaysia

**Keywords:** trigeminovascular system, GABA, α6GABA_A_R, positive allosteric modulator, trigeminal ganglia, pyrazoloquinolinone

## Abstract

**Introduction:**

The α6 subunit-containing GABA_A_ receptors (α6GABA_A_Rs) are highly expressed in the trigeminal ganglia (TG), the sensory hub of the trigeminovascular system (TGVS). Hypo-GABAergic transmission in the TG was reported to contribute to migraine-related behavioral and histopathological phenotypes. Previously, we found that Compound 6, an α6GABA_A_R-selective positive allosteric modulator (PAM), significantly alleviated TGVS activation-induced peripheral and central sensitization in a capsaicin-induced migraine-mimicking model.

**Methods:**

Here, we tested whether the deuterated analogues of Compound 6, namely DK-1-56-1 and RV-I-29, known to have longer half-lives than the parent compound, can exert a similar therapeutic effect in the same model. The activation of TGVS was triggered by intra-cisternal (*i.c.*) instillation of capsaicin in male Wistar rats. Centrally, *i.c.* capsaicin increased the quantity of c-Fos-immunoreactive (c-Fos-ir) neurons in the trigeminal cervical complex (TCC). Peripherally, it increased the calcitonin gene-related peptide immunoreactivity (CGRP-ir) in TG, and caused CGRP release, leading to CGRP depletion in the dura mater.

**Results:**

DK-I-56-1 and RV-I-29, administered intraperitoneally (*i.p.*), significantly ameliorated the TCC neuronal activation, TG CGRP-ir elevation, and dural CGRP depletion induced by capsaicin, with DK-I-56-1 demonstrating better efficacy. The therapeutic effects of 3 mg/kg DK-I-56-1 are comparable to that of 30 mg/kg topiramate. Notably, *i.p.* administered furosemide, a blood-brain-barrier impermeable α6GABA_A_R-selective antagonist, prevented the effects of DK-I-56-1 and RV-I-29. Lastly, orally administered DK-I-56-1 has a similar pharmacological effect.

**Discussion:**

These results suggest that DK-I-56-1 is a promising candidate for novel migraine pharmacotherapy, through positively modulating TG α6GABA_A_Rs to inhibit TGVS activation, with relatively favourable pharmacokinetic properties.

## 1 Introduction

The current pharmacotherapy for migraine provides pain relief to many patients, but it is associated with several drawbacks, including refractory responses, adverse effects, low compliance, etc. (Puledda et al., 2023). Conventional oral-active anti-migraine agents are commonly prescribed but often met with low patient compliance due to the frequency of dosing. The calcitonin gene-related peptide (CGRP)- and CGRP receptor-targeting protein drugs and onabotulinumtoxin A are available in monthly or quaternary doses. Although non-peptide CGRP receptor antagonists, are recently available, the high medical cost and long-term safety, especially in patients with cardiovascular diseases, remain important clinical considerations (Favoni et al., 2019; Lampl et al., 2023). Thus, orally administered drugs still remain the most cost-effective preventive medication for migraine (Mistry et al., 2023; Karsan and Goadsby, 2022), e.g., NSAIDs, triptans, antiepileptics, etc. However, current oral antimigraine agents often met with refractory responses and undesirable side effects (Bentivegna et al., 2024; Tronvik et al., 2024). These call for continuous research into novel treatments that may potentially address these concerns while providing an optimal anti-migraine effect.

One of the pathophysiological hallmarks of migraine is the hyperactivity of the trigeminovascular system (TGVS) (Puledda et al., 2023), which encompasses the trigeminal ganglia (TG) that transmit sensory information about the meninges and cerebral vessels to the central nervous system (Terrier et al., 2021). CGRP is abundantly expressed in TG neurons and released during migraine attack to induce dural vasodilatation and inflammation. Using the intermittent repeated nitroglycerin (irNTG)-induced chronic migraine model in mice, we have previously reported that hypofunction of GABAergic transmission in the TG may contribute to migraine-mimicking behaviors, while the expression of α6 subunit-containing GABA_A_ receptors (α6GABA_A_Rs) in TG was not affected by repeated NTG administration (Tzeng et al., 2021). By harnessing this unique pathophysiological feature, we demonstrated that α6GABA_A_R-selective positive allosteric modulators (PAMs) significantly alleviated the NTG-induced migraine-mimicking grimaces in mice in a manner prevented by furosemide, an α6GABA_A_R-selective antagonist (Tzeng et al., 2021). In a capsaicin-induced TGVS activation model in rats that mimics the histopathological features of migraine, we also found the α6GABA_A_R-selective PAM significantly attenuated TGVS activation-induced peripheral and central sensitization in a furosemide-sensitive manner (Fan et al., 2018). Taken together, our previous studies strongly suggested that TG, the sensory hub of TGVS, is the main target site for α6GABA_A_R PAMs to ameliorate the migraine-mimicking phenotypes.

**FIGURE 1:**
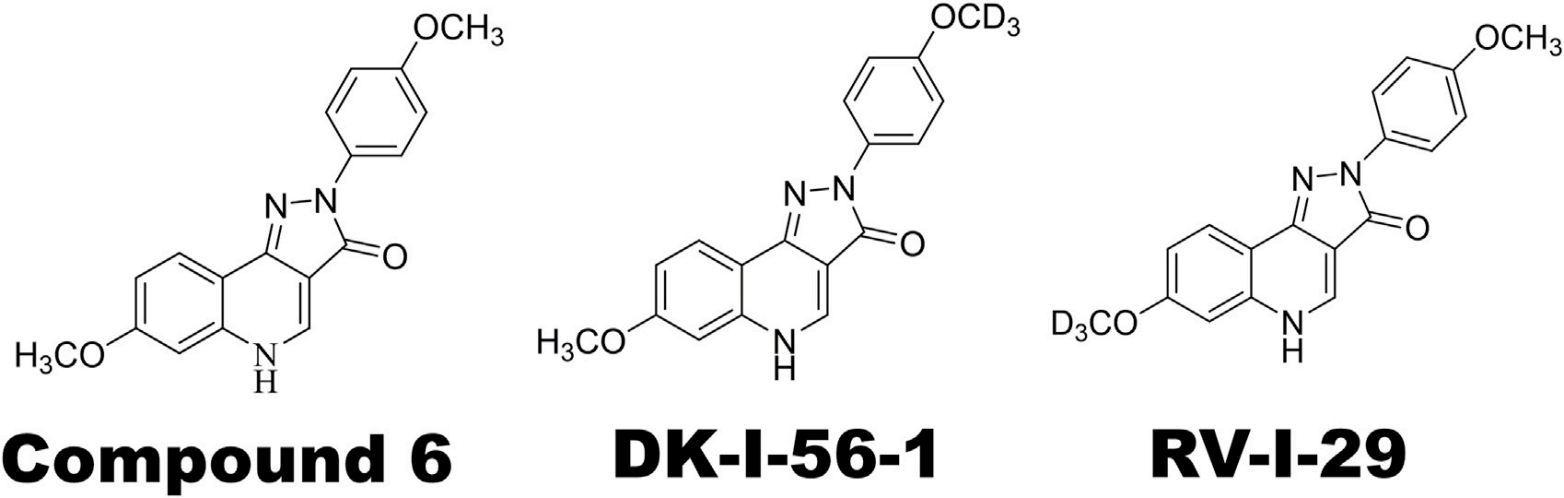
Chemical structures of α6GABA_A_R-selective positive allosteric modulators (PAMs) used in this study. Note that DK-I-56-1 and RV-I-29 are deuterated analogues of Compound 6.

The main treatment lead, Compound 6 (Figure 1), the highest efficacious compound in the first series of pyrazoloquinolinones (PQs) reported as PAM selective for α6GABA_A_R, has a relatively short plasma half-life (Knutson et al., 2018; Varagic et al., 2013a). Various deuterated derivatives of Compound 6 have been introduced, of which DK-I-56-1 and RV-I-29, deuterated derivatives at the methoxy groups of D-ring and A-ring, respectively (Figure 1), are noteworthy. They retain the high selectivity towards α6GABA_A_Rs while have longer half-lives as compared with Compound 6 (Knutson et al., 2018). In the present study, we studied the effects of DK-I-56-1 and RV-I-29 using the capsaicin-induced TGVS activation model, in comparison with their parent compound, Compound 6.

## 2 Materials and methods

### 2.1 Animals

Male Wistar rats, 8–10 weeks old and weighing 250–300 g, were purchased from BioLASCO (Taiwan Co., Ltd.). A total of 76 rats were used in this study. The rats were kept in an animal facility with a 12 h light/dark cycle and had free access to food and water. The Institutional Animal Care and Use Committee of the National Taiwan University College of Medicine approved the animal care and experimentation protocols (Approval code 20160170).

### 2.2 Intracisternal (i.c.) administration of capsaicin

**SCHEME 1:**
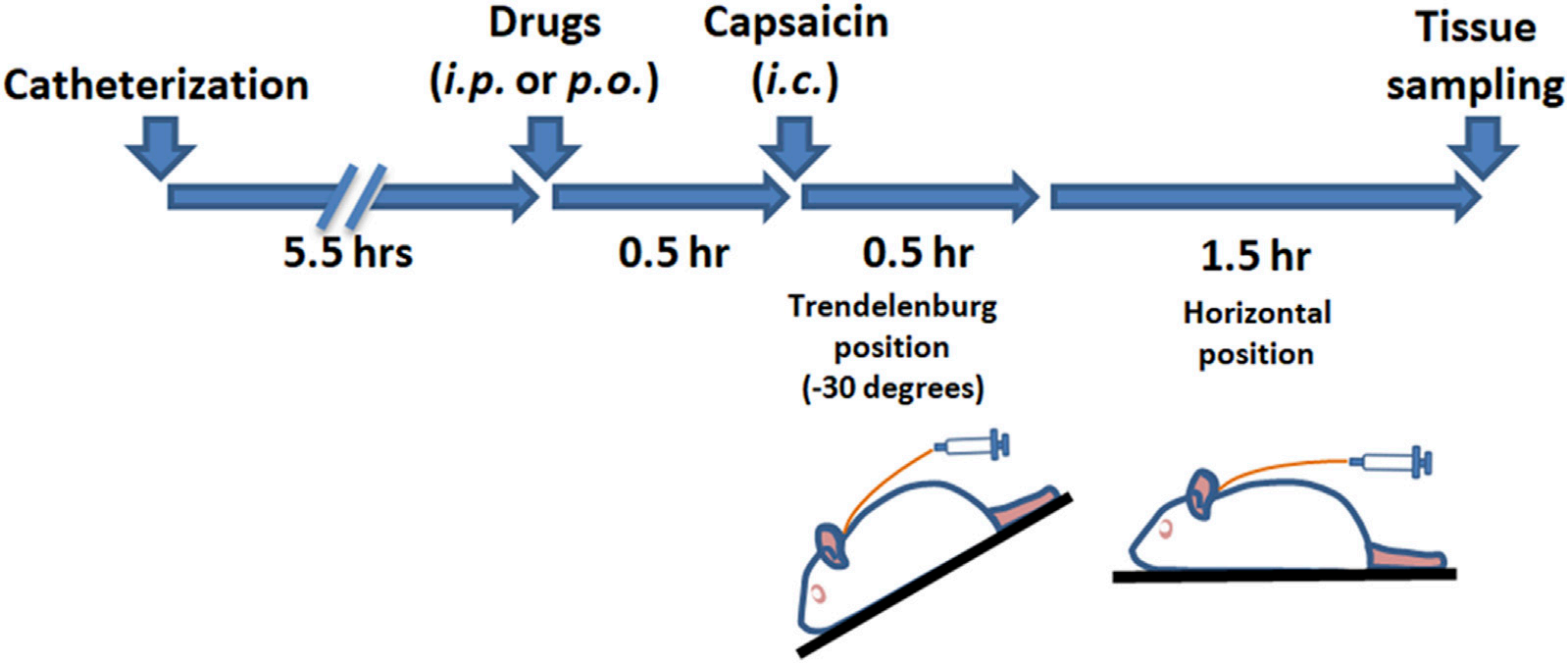
The timeline of the experimental procedure.

Activation of the TGVS was achieved via intracisternal (i.c.) administration of capsaicin (Fan et al., 2018; Fan et al., 2012; Huang et al., 2018), following previously established methods. As outlined in Scheme 1, rats were anesthetized with chloral hydrate (400 mg/kg for induction and 100 mg/kg for maintenance, instilled intraperitoneally). A catheter (PE-10, SIMS Portex Ltd., Hythe, United Kingdom) was inserted 3 mm into the cisterna magna of each anesthetized rat. The rats were then placed in a prone position for 5.5 h. Capsaicin (10 nmol, 100 μL) or its vehicle for the sham group was instilled into the cisterna magna via the catheter over 1 min, followed by positioning the rats in a reverse Trendelenburg (−30°) for 30 min to ensure proper distribution of the capsaicin in the subarachnoid space. The rats were then returned to the prone position for another 90 min. DK-I-56-1, RV-I-29, or a vehicle was administered via intraperitoneal (*i.p.*) injection or oral gavage 30 min prior to capsaicin instillation. Furosemide, an α6GABA_A_R antagonist (Agunu et al., 2005; Korpi and Luddens, 1997), or its vehicle, was co-administered with DK-I-56-1 or RV-I-29 as needed. The administration volume was 0.5 mL per 300 g of body weight.

Two hours post capsaicin instillation, the rats were euthanized and subsequently perfused with paraformaldehyde (4%) via the ascending aorta for further immunostaining procedures (Fan et al., 2018; Fan et al., 2012; Huang et al., 2018).

### 2.3 Immunohistochemistry of cFos protein in trigeminal cervical complex (TCC) sections

The TCC sections with c-Fos immunohistochemistry were prepared following established protocols (Fan et al., 2018; Fan et al., 2012; Huang et al., 2018). In summary, the brainstem along with the cervical cord attached was serially sectioned at 50 μm intervals using a cryostat (LEICA CM3050S, Nussloch, Germany) from 1 mm rostral to the obex down to the C6 of the spinal cord. The sections located at +0.6, −1.2, and −9 mm from obex in rats were specifically collected for c-Fos immunostaining. The total count of c-Fos immunoreactive (c-Fos-ir) TCC neurons was determined using the formula derived previously: 16(N1 + N2)/2 + 53(N2 + N3)/2, where N1, N2, and N3 represent the c-Fos-ir neuronal counts at the respective distances from the obex (−9 mm, −1.2 mm, and +0.6 mm).

The c-Fos protein immunohistochemistry was performed using the avidin-biotin method, following established protocols (Fan et al., 2012). A polyclonal antibody of rabbits against c-Fos with 1:7,000 dilution (Calbiochem, San Diego, CA, United States), biotinylated anti-rabbit IgG 1:200 (Vector Labs, Burlingame, CA, United States), and horseradish peroxidase avidin D 1:500 (Vector Labs, Burlingame, CA, United States) were utilized. Visualization of immunoreactions was achieved using the DAB Reagent Kit (KPL, Gaithersburg, MD, United States). Neurons expressing c-Fos, indicated by nuclei stained, were quantified using a microscope (Olympus BX51, Essex, United Kingdom). An investigator who was blinded to the treatment groups conducted the data analysis.

### 2.4 Trigeminal ganglia (TG) slice sections and CGRP immunofluorescence quantification

The preparation of TG sections and the quantification of CGRP immunofluorescence were carried out following established procedures (Fan et al., 2018; Fan et al., 2012; Huang et al., 2018). Briefly, 2 TG tissues were dissected from each rat and subsequently sectioned at a thickness of 50 μm using a microtome (LEICA RM2245, Nussloch, Germany). We obstained nine sections from the central region of either the right or left TG from each rat. Subsequently, every third section was selected for CGRP immunofluorescence analysis, resulting in data collection from 3 TG sections per rat.

The quantification of immunofluorescence followed established procedures (Fan et al., 2018; Fan et al., 2012; Huang et al., 2018). Sections were exposed to a primary antibody of rabbit targeting CGRP with 1:200 dilution (EMD Millipore, Burlington, MA, United States) and subsequently treated with a solution containing fluorescein-conjugated goat anti-rabbit IgG 1:50 (Vector Labs, Burlingame, CA, United States) as secondary antibody. Afterward, we mounted sections onto microscope slides using Aqua Poly/Mount (Polyscience Inc., Warrington, PA, United States) and observed the tissue sections with an inverted light microscope (Zeiss Axio Observer, D1; Carl Zeiss, Jena, Germany). Analysis was performed on fields of TG at ×100 magnification using ImageJ software. The intensity of CGRP expression was determined based on the immunoreactivity, represented by the optical density multiplied by the activated areas in each image.

### 2.5 Quantification of CGRP-ir in dura mater immunoreactivity

In accordance with previous research (Fan et al., 2018; Fan et al., 2012; Huang et al., 2018), the dura mater of rats was excised from the skull and processed for CGRP immunohistochemical staining. Briefly, six corresponding locations on the dura were identified and observed with an inverted microscope (ZEISS Axio Observer.D1, Jena, Germany). For each rat, we quantified total lengths of positively stained segmented lines within the field (×100 magnification) in pixels using ImageJ software.

The immunostaining for CGRP was conducted using the avidin-biotin method, following a protocol for staining c-Fos. However, this process involved a 30-min blocking incubation period, an anti-CGRP polyclonal antibody of rabbit in 1:1,000 of dilution (Calbiochem, San Diego, CA, United States), and horseradish peroxidase avidin D 1:200 (Vector Labs, Burlingame, CA, United States).

### 2.6 Chemicals and drugs

We prepared capsaicin (Sigma Chemical, St. Louis, MO, United States) solution by mixing with the vehicle containing 10% ethanol and 10% Tween 80. After 5 min sonication, the capsaisin solution was then diluted (1:100) in an aCSF, which was used as a stock solution and stored at 4°C. The aCSF contained the components (in mm): 117 NaCl, 4.5 kC l, 2.5 CaCl_2_, 1.2 MgCl_2_, 1.2 NaH_2_PO_4_, 25 NaHCO_3_, and 11.4 dextrose bubbled with 95% O_2_/5% CO_2_, pH 7.4. The α6GABA_A_R PAMs (Figure 1), Compound 6 [7-methoxy-2-(4-methoxyphenyl)-2,5-dihydro-3-H-pyrazolo[4,3-c]-quinolin-3-one], DK-I-56–1 [7-methoxy-2-(4-methoxy-d3-phenyl)-2,5-dihydro-3-H-pyrazolo-[4,3-c]quinolin-3-one] and RV-I-29 [7-(methoxy-d3)-2-(4-methoxyphenyl)-2,5-dihydro-3H-pyrazolo[4,3-c]quinolin-3-one] were synthesized as reported previously (Knutson et al., 2018). Furosemide was purchased from Sigma-Aldrich (St. Louis, United States). All PQ compounds were dissolved in a vehicle containing 20% DMSO, 20% Cremophor^®^ EL (polyoxyethylene castor, Sigma-Aldrich) and 60% normal saline.

### 2.7 Statistical analysis

Data were presented as median and interquartile ranges. The Kruskal–Wallis test was used to assess group differeneces. Subsequently, comparisons between the tested group and the capsaicin-treated group (Family-1, 3, 5 Supplementary Tables S1–S3) or the sham-control group (Family-2, 4, 6, Supplementary Tables S1–S3) were made using the Mann-Whitney U test, followed by the Benjamini–Hochberg correction (BHC), which is appropriate correction to control the false positive rate resulting from multiple comparisons (McDonald, 2014). For each comparison pair within a family, the p-value was ranked. A statistically significant difference was determined when the p-value was smaller than the BHC value, calculated as 0.05 multiplied by i/m, where m represents the total number of comparisons within each family, and i denotes the rank of the p-value in the family. The n numbers indicate the number of rats tested in each treatment group. TCC, TG, and dura mater samples were prepared from each rat for analysis. Statistical analyses were conducted with IBM SPSS Statistics 20 for Windows.

## 3 Results

**FIGURE 2:**
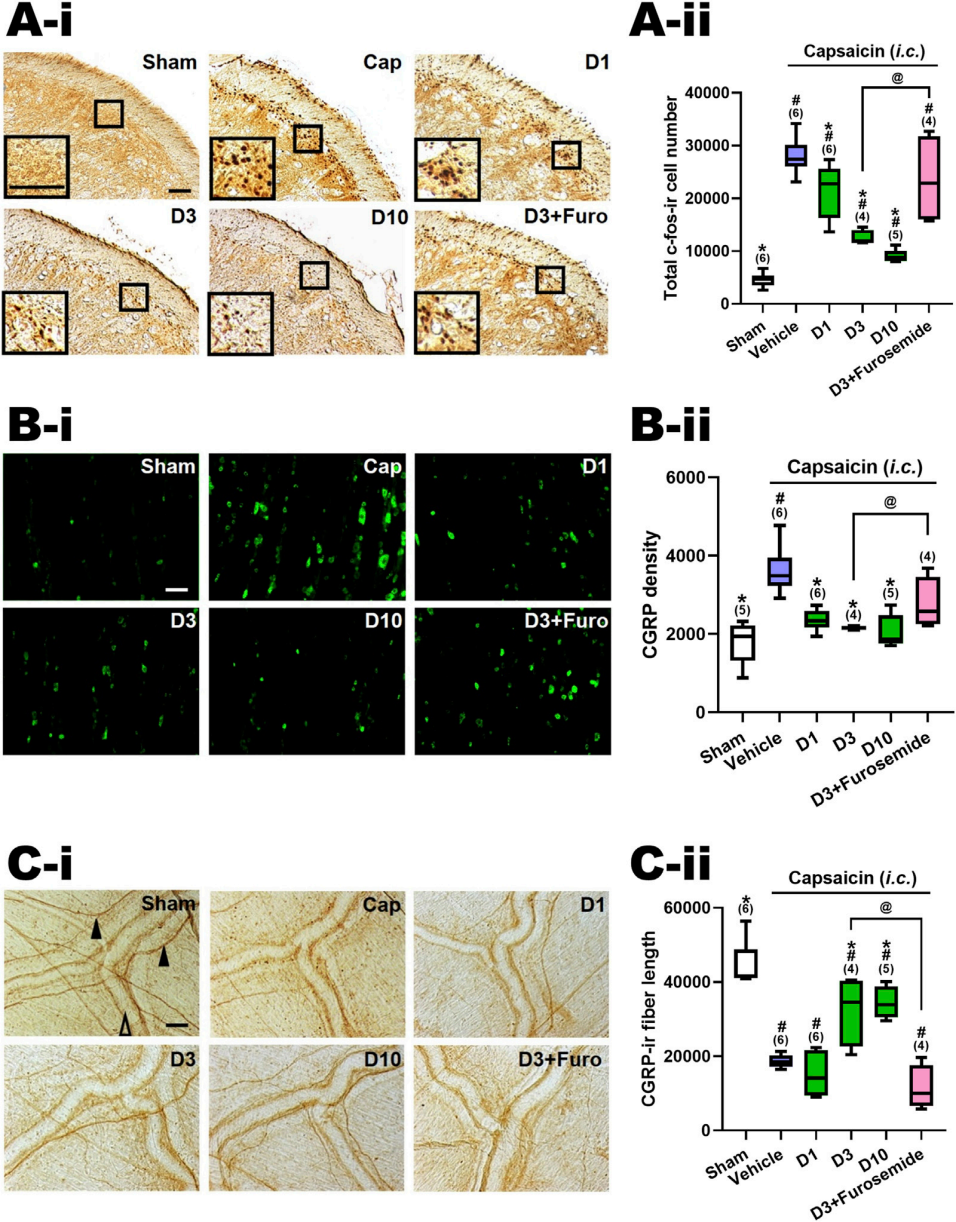
Effects of DK-I-56-1 (*i.p.*) on capsaicin-induced neuronal activation and anti-α6GABA_A_R effects of furosemide in the trigeminal cervical complex (TCC), trigeminal ganglia (TG) and dura mater. Immunohistograms **(A-i)** and the total number of activated neurons **(A-ii)**, i.e. c-Fos-immunoreactive (c-Fos-ir) neurons, in the TCC of rats having received intracisternal (*i.c.*) instillation of 10 nmol capsaicin in the group pretreated with *i.p.* injection of DK-I-56-1 at 1 (D1), 3 (D3) or 10 (D10) mg/kg, as well as with 3 mg/kg DK with 20 mg/kg furosemide (*i.p.*) (D3+Furosemide). The sham group received *ic.* instillation of the vehicle instead of capsaicin (Sham). A close-up image (inset) of c-Fos-containing TCC neurons **(A-i)** is shown in each treatment group. Note that DK-I-56-1 at 1, 3 or 10 mg/kg significantly attenuates capsaicin-induced TCC neuronal activation. The inhibitory effect of DK-I-56-1 in the TCC was significantly reversed by furosemide, a highly selective allosteric inhibitor for α6GABA_A_Rs. **(B-i)** DK-I-56-1 at 1, 3 or 10 mg/kg significantly prevents capsaicin-induced CGRP-ir in TG. The capsaicin-induced CGRP-ir inhibited by DK-I-56-1 at 3 mg/kg is significantly reversed by furosemide **(B-ii)**. **(C-i)** DK-I-56-1 at 3 or 10, but not 1, mg/kg significantly suppressed capsaicin-induced depletion of dura CGRP-ir. The capsaicin-induced CGRP depletion inhibited by DK at 3 mg/kg is significantly reversed by furosemide **(C-ii)**. Scale bar: **(A-i)** 500 um (a); 100 um (b). (**B-i**) 500 um (a and b); 250 um (c). (**C-i**) 1,000 um (a); 400 um (b). 100 um (c). CC: central canal; Sp5C: spinal trigeminal nucleus caudalis. In each rat, total CGRP-ir fluorescence in 3 TG sections was measured. Shown is the average CGRP-ir fluorescence of all tested rats. The length of CGRP-ir nerve fibers (arrowhead), stained by immunohistochemistry, in the dura mater was quantified by ImageJ. The total length of CGRP-ir fibers (in pixel) in six fixed comparable areas of the dura mater in each rat was collected. Filled arrowhead: CGRP-ir nerve fiber; Empty arrowhead: dural vessel. Data are presented as median and interquartile ranges. The *n* number shown in parentheses is the number of tested rats. *p < 0.0125 x i, vs. the Vehicle (Capsaicin) group; ^#^ *p* < 0.01 x i, *vs.* the Sham group; i: the rank of p value (Supplementary Tables S1, S2). (Kruskal–Wallis test followed by *post hoc* Mann-Whitney U test with Benjamin-Hochberg correction). ^@^ *p* < 0.05 by Mann-Whitney U test.

**FIGURE 3:**
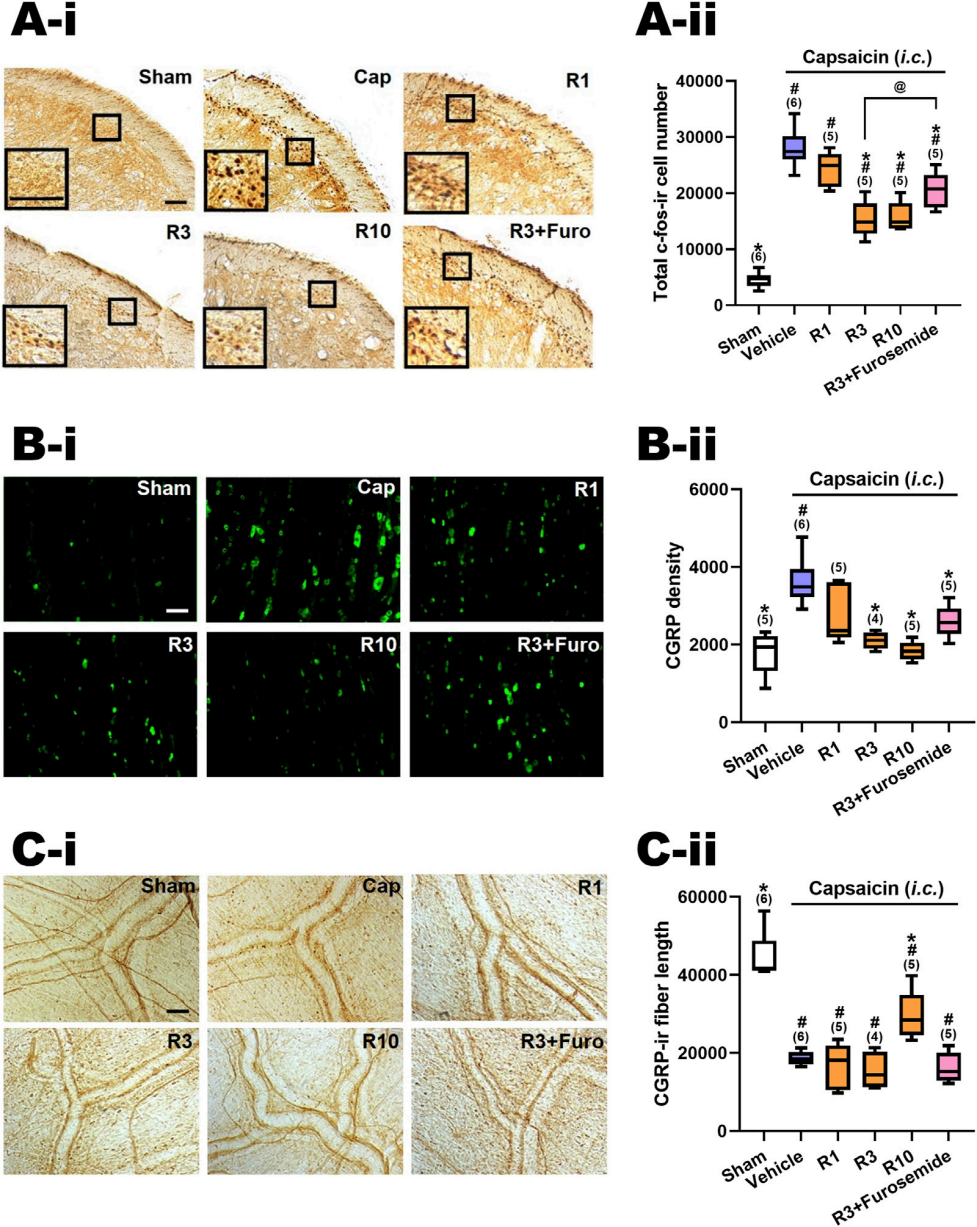
Effects of RV-I-29 (*i.p.*) on capsaicin-induced neuronal activation and anti-α6GABA_A_R effects of furosemide in the trigemino-cervical complex (TCC), trigeminal ganglia (TG) and dura mater. Immunohistograms **(A-i)** and **(A-ii)** c-Fos-immunoreactive (c-Fos-ir) neurons in the TCC of rats having received intracisternal (*i.c.*) instillation of 10 nmol capsaicin in the group pretreated with *i.p.* injection of RV-I-29 at 1 (R1), 3 (R3) or 10 (R10) mg/kg, as well as with 3 mg/kg RV plus 20 mg/kg furosemide (*i.p.*) (R3+Furosemide). The sham group received *i.c.* instillation of the vehicle instead of capsaicin (Sham). A close-up image (inset) of c-Fos-containing TCC neurons **(A-i)** is shown in each treatment group. Note that RV-I-29 at 3 or 10, but not 1 mg/kg significantly attenuates capsaicin-induced TCC neuronal activation. The inhibitory effect of RV in the TCC was significantly, but not fully, reversed by furosemide. **(B-i)** RV-I-29 at 3 or 10, but not 1 mg/kg significantly prevents capsaicin-induced CGRP-ir in TG **(B-ii)**. The capsaicin-induced CGRP-ir inhibited by RV-I-29 at 3 mg/kg is partially reversed by furosemide. **(C-i)** RV-I-29 at 10, but not one or 3, mg/kg significantly suppressed capsaicin-induced depletion of dura CGRP-ir **(C-ii)**. Scale bar, data presentation and statistical analyses are the same as in Figure 2. Note that the immunohistograms of Cap and Sham groups are the same as in Figure 2.

In line with prior research [13, 17], *i.c.* capsaicin (10 nmol) administration in rats notably stimulated both central and peripheral ends of the TGVS. It elicited neuronal activation in the TCC, centrally, as evidenced by the elevated count of c-Fos-ir neurons (Cap vs. Sham, Figures 2A, 3A-i). Peripherally, instillation of capsaicin resulted in heightened CGRP-ir in the TG (Cap vs. Sham, Figures 2B, 3B-i) and CGRP excretion from trigeminal nerve terminals, as indicated by the reduction in CGRP-ir observed in the dura mater (Cap vs. Sham, Figures 2C, 3C-i).

### 3.1 DK-I-56-1 reduced capsaicin-induced neuronal activation in TCC

Figures 2A-ii illustrates the total count of c-Fos-ir TCC neurons in various groups of rats, including those that received *i.c.* capsaicin instillation, pretreated with DK-I-56-1 (at doses of 1, 3, and 10 mg/kg, *i.p.*), its vehicle (*i.p.*), and the sham control group that received *i.c.* capsaicin vehicle only. Significant differences were observed among the groups (*p* < 0.001 by Kruskal–Wallis test). We compared each treatment group with the capsaicin group (Family 1, Supplementary Table S1) or with the sham group (Family 2, Supplementary Table S1) using The Mann-Whitney U test followed by the Benjamini–Hochberg correction.Four or five comparisons were conducted within each family totally.

In our prior investigation [10, 13, 14], it was observed that rats subjected to capsaicin instillation exhibited a notable rise in the c-Fos-ir TCC neuron counts in comparison with the sham group (*p* < 0.005, Cap vs. Sham, Supplementary Table S2; Figure 2B). Upon pretreatment with DK-I-56-1, a significant reduction in the capsaicin-induced c-Fos-ir TCC neuron number was evident at doses of one (*p* = 0.025, D1 vs. Cap, Figure 2A-ii), 3 (*p* = 0.011, D3 vs. Cap, Figure 2B) and 10 (*p* = 0.006, D10 vs. Cap, Figure 2A-ii) mg/kg in comparison with the capsaicin group treated solely with the vehicle *i.p.* (Figure 2A-ii; Supplementary Table S1). The potency of DK-I-56-1 appeared to increase in tandem with dosage escalation in a dose-dependent manner (*p* = 0.019, 3 *vs.* 1 mg/kg; *p* = 0.016, 10 *vs.* 3 mg/kg, Figure 2A-ii). These findings indicate that even at a low *i.p.* dose of 1 mg/kg, DK-I-56-1 significantly mitigates capsaicin-induced TCC neuronal activation. Nonetheless, DK-I-56-1, even at a high dose up to 10 mg/kg, failed to reduce the activated TCC neuron number to the level observed in sham group (*p* = 0.004, 0.011, and 0.006 for 1 (D1), 3 (D3) and 10 (D3) mg/kg DK-I-56-1 vs. Sham, respectively; Figure 2A-ii, Supplementary Table S1).

We investigated the impact of *i.p*. furosemide, an antagonist of α6GABA_A_Rs (Korpi and Luddens, 1997), on the inhibition of TCC neuronal activation by capsaicin induced by DK-I-56-1 (3 mg/kg) (D3+Furosemide, Figure 2A-ii). The suppressive effect of DK-I-56-1 on TCC activity was notably reversed by furosemide. The number of c-fos-ir cells in the group treated with DK-I-56-1 (3 mg/kg) combined with furosemide (20 mg/kg, *i.p.*) was significantly different from the group treated solely with DK-I-56-1 (*p* = 0.021, D3 vs. D3+Furosemide, Figure 2A-ii), reaching levels comparable to those seen in the disease group (*p* = 0.522, D3+Furosemide vs. Cap, Figure 2A-ii). These findings suggest that the systemic administration of furosemide counteracts the inhibition of DK-I-56-1 on capsaicin induced TCC neuronal activation completely.

### 3.2 DK-I-56-1 attenuated TG capsaicin-induced CGRP-ir

Figure 2B-ii illustrates the levels of CGRP-ir in the TG across different experimental groups, as depicted in Figure 2A-ii. There were significant differences among groups (*p* = 0.002 by Kruskal–Wallis test). The Mann-Whitney U test with the Benjamini–Hochberg correction revealed that the CGRP-ir in the TG of the capsaicin *i.c.* treated group was notably elevated in comparison with the sham (*p* = 0.006, Cap vs. Sham, Figure 2B-ii). This indicates a significant increase in TG CGRP-ir induced by *i.c.* capsaicin, consistent with our previous findings (Fan et al., 2018; Fan et al., 2012; Huang et al., 2018). In line with these observations, DK-I-56–1 was found to suppress TG CGRP-ir at doses of one (*p* = 0.004, D1 vs. Cap, Figure 2B-ii), 3 (*p* = 0.011, D3 vs. Cap, Figure 2B-ii) and 10 mg/kg (*p* = 0.006, D10 vs. Cap, Figure 2B-ii), relative to the capsaicin group. Remarkably, the levels of TG CGRP-ir were reduced to those akin to the sham group by DK-I-56–1 at doses of 1, 3, or 10 mg/kg (*p* = 0.068, 0.221 and 0.754 for 1 (D1), 3 (D3) and 10 (D10) mg/kg DK-I-56-1 vs. Sham, respectively; Figure 2B-ii, Supplementary Table S1). Specifically, DK-I-56-1 at 1 mg/kg completely abolished the increase in TG CGRP-ir induced by capsaicin, with no further suppression observed upon increasing the dose to 3 or 10 mg/kg (Figure 2B-ii).

Consistent with the findings in the TCC, furosemide substantially counteracted the inhibitory effects induced by DK-I-56-1 in the TG. The CGRP density in the TG pretreated with DK-I-56-1 (3 mg/kg) along with furosemide was notably restored, reaching levels insignificantly different from those observed in the capsaicin *i.c.* treated group (*p* = 0.136; C3+Furosemide vs. Cap, Figure 2B-ii). Furthermore, the TG CGRP density was significantly increased in the group pretreated with DK-I-56-1 plus furosemide compared to those treated solely with DK-I-56-1 at 3 mg/kg (*p* = 0.021, D3 vs. D3+Furosemide, Figure 2B-ii). These results indicate that systemic administration of furosemide significantly influences the inhibitory effect induced by DK-I-56-1 on TG CGRP-ir following exposure to capsaicin.

### 3.3 DK-I-56-1 reversed capsaicin-induced depletion of CGRP-ir in dura mater

Figure 2C-ii displays the CGRP-ir in the dura mater across different treatment groups, quantified by the total length of CGRP-ir nerve fibers. There were significant differences among groups (p< 0.001 by Kruskal–Wallis test). A notable depletion of CGRP-ir in the dura mater following *i.c.* capsaicin instillation was observed (Cap vs. *S*ham, Figure 2C-ii), consistent with previous findings (Fan et al., 2012) using the Mann-Whitney U test with the Benjamini–Hochberg correction. This was evidenced by the reduced CGRP-ir nerve fiber length in the capsaicin-treated group compared to the sham group (p< 0.005, Cap vs. Sham, Figure 2C-ii). Importantly, DK-I-56-1 treatment significantly rescued the capsaicin -induced CGRP depletion, as evidenced by the substantial but not complete restoration of CGRP-ir fiber length in the dura mater of groups pretreated with DK-I-56-1 at 3 (*p* = 0.019, D3 vs. -Cap; *p* = 0.011, D3 vs. Sham, Figure 2C-ii) and 10 mg/kg (*p* = 0.006, D10 vs. Cap; *p* = 0.006, D10 vs. Sham, Figure 2C-ii), though not at 1 mg/kg (*p* = 0.337, D1 vs. Cap, Figure 2C-ii).

The CGRP-ir nerve fiber length in the dura mater was notably reduced in the group pretreated with DK-I-56-1 plus furosemide in comparison with DK-I-56-1 at 3 mg/kg (*p* = 0.021, D3 vs. D3+Furosemide, Figure 2C-ii). These findings imply that systemic administration of furosemide effectively reverses the inhibitory effect induced by DK-I-56-1 on CGRP-ir depletion caused by capsaicin in the dura mater.

### 3.4 RV-I-29 attenuated capsaicin-induced TCC neuronal activation

Figure 2C-ii illustrates the effects of RV-I-29, another methoxy deuterated derivative of Compound 6 distinct from DK-I-56-1 on *i.c.* capsaicin-treated rats. Figure 3A-ii displays the total c-Fos-ir TCC neuronal number in groups that received capsaicin *i.c.,* pretreated with RV-I-29, along with the sham control group that received *i.c.* capsaicin vehicle instillation only. Significant differences were observed among the groups (*p* < 0.001 by Kruskal–Wallis test). We compared each treatment group with the capsaicin group (Family 3, Supplementary Table S2) or with the sham group (Supplementary Table S2, Family 4) by the Mann-Whitney U test with the Benjamini–Hochberg correction. Each family had four or five comparisons conducted.

In rats pretreated with RV-I-29, the count of capsaicin induced c-Fos-ir TCC neurons was significantly diminished at doses of 3 (*p* = 0.006, R3 vs. Cap, Figure 3A-ii) and 10 (*p* = 0.006, R10 vs. Cap, Figure 3A-ii) mg/kg, but not 1 mg/kg (*p* = 1.0, R1 vs. Cap, Figure 3A-ii), in comparison with the capsaicin group pretreated with vehicle (Figure 3A-ii; Supplementary Table S2). These findings indicate that RV-I-29 substantially lessens capsaicin-induced neuronal activation in TCC at doses exceeding 1 mg/kg. However, akin to DK-I-56-1, RV-I-29, whether administered at 3 or 10 mg/kg, did not lower activated TCC neuronal number to the extent observed in the sham group (*p* = 0.006 and 0.006 for 3 (R3) and 10 (R10) mg/kg RV vs. Sham, respectively; Figure 3A-ii, Supplementary Table S2).

We also investigated the impact of systemically administered furosemide *i.p.* (20 mg/kg) on the suppression of neuronal activation induced by capsaicin in TCC when pretreated with RV-I-29 (3 mg/kg, *i.p.*) (R3+Furosemide, Figure 3A-ii). Significantly, furosemide was able to reverse the inhibitory effect of RV-I-29 on TCC activation. There was a notable difference in the c-fos-ir cell count between the groups treated with RV-I-29 (3 mg/kg) with and without furosemide (*p* = 0.028, R3 vs. R3+Furosemide, Figure 3A-ii). However, it's important to note that while furosemide partially restored the c-fos-ir cell count, it did not completely reach the level observed in the capsaicin group (*p* = 0.011, R3+Furosemide vs. Cap, Figure 3A-ii). This result suggests that furosemide administered systemically can partially counteract the RV-I-29 induced inhibitory effect on the TCC neuronal activation triggered by capsaicin.

### 3.5 RV-I-29 attenuated capsaicin-induced CGRP-ir in TG

Figure 3B–ii illustrates the CGRP-ir in the TG across different experimental groups, mirroring the presentation in Figure 3A-ii. Significant differences were observed among these groups (p< 0.001 by Kruskal–Wallis test). The CGRP-ir in TG was notably suppressed by RV-I-29 at doses of 3 (*p* = 0.011, R3 vs. Cap, Figure 3C) and 10 mg/kg (*p* = 0.006, R10 vs. Cap, Figure 3C), but not 1 mg/kg (*p* = 0.144, R1 *vs.* Cap, Figure 3B–ii), compared to the capsaicin group pretreated with *i.p.* vehicle. The levels of CGRP-ir in TG were reduced to those comparable to sham group by doses of 3 or 10 mg/kg of RV-I-29, but not by the 1 mg/kg dose (*p* = 0.047, 0.221 and 0.602 for 1 (R1), 3 (R3) and 10 (R10) mg/kg RV vs. Sham, respectively; Figure 3C, Supplementary Table S2).

Furosemide showed a tendency to reverse the inhibitory effects induced by RV-I-29 in the TG, although this effect was not statistically significant. The TG CGRP density of rats pretreated with RV-I-29 (3 mg/kg) along with furosemide was insignificantly restored in comparison with the capsaicin-treated group (*p* = 0.011, R3+Furosemide vs. Cap, Figure 3B–ii). Additionally, there was no significant difference in CGRP density between the group pretreated with RV-I-29 plus furosemide and those treated solely with RV-I-29 (3 mg/kg) (*p* = 0.086, R3 vs. R3+Furosemide, Figure 3B–ii). These findings suggest systemic instillation of furosemide did not have a significant impact on the inhibitory effect induced by RV-I-29 on TG CGRP-ir following exposure to capsaicin.

### 3.6 RV-I-29 reversed capsaicin-induced reduction of CGRP-ir in the dura mater

Figure 3C–ii displays the CGRP-ir levels, measured by the length of CGRP-ir nerve fibers, in the dura mater of rats across different treatment groups. The differences were statistically significant among groups (*p* = 0.001 by Kruskal–Wallis test). RV-I-29 notably countered the CGRP depletion induced by capsaicin, as evidenced by the partial restoration of CGRP-ir fiber length in the dura mater of rats pretreated with 10 mg/kg of RV-I-29 (*p* = 0.006, R10 vs. Cap; *p* = 0.006, R10 vs. Sham, Figure 3C–ii), but this effect was not observed at the one (*p* = 0.855, R1 vs*.* Cap, Figure 3C–ii) or 3 mg/kg (*p* = 0.286, R3 vs. Cap, Figure 3C–ii) dosage.

### 3.7 Comparison in effects of the deuterated compounds, DK-I-56-1 and RV-I-29, with its parent compound, compound 6

**FIGURE 4:**
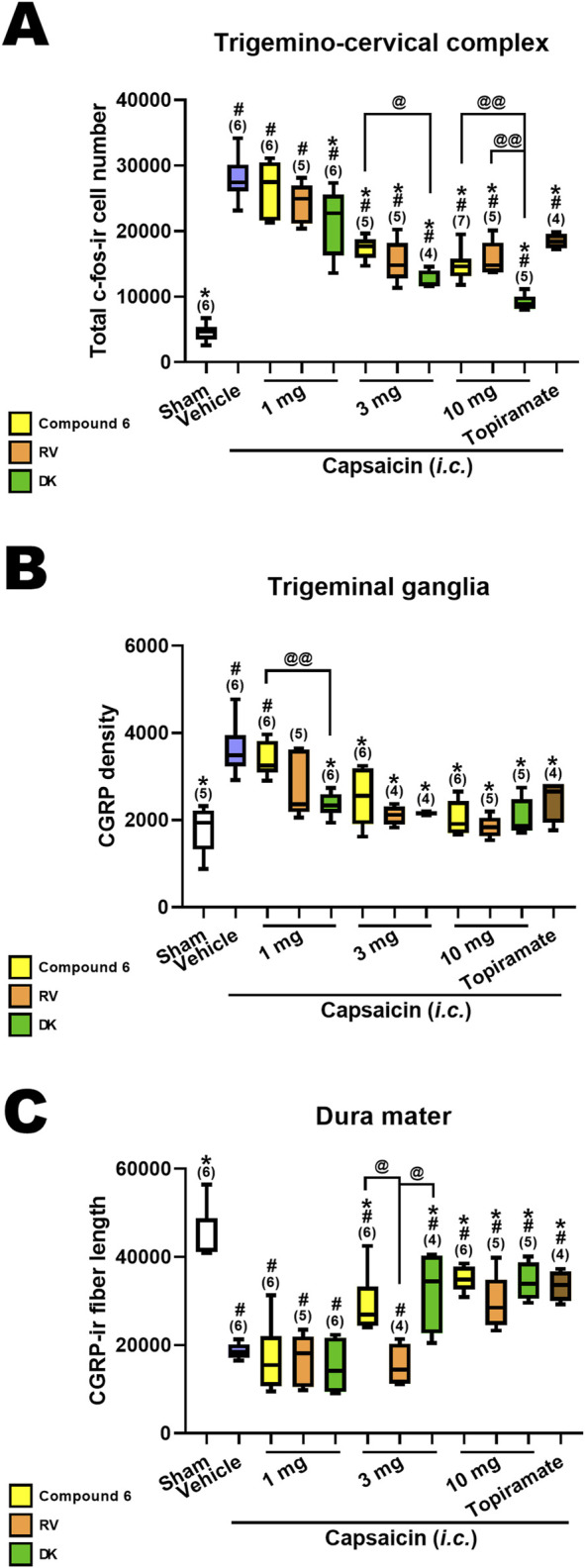
Comparison in effects of Compound 6, RV-I-29 and DK-I-56-1 (*i.p.*) on capsaicin-induced neuronal activation as well as topiramate in the trigemino-cervical complex (TCC), trigeminal ganglia (TG) and dura mater. **(A)** c-Fos-immunoreactive (c-Fos-ir) neurons in the TCC of rats having received intracisternal (*i.c.*) instillation of 10 nmol capsaicin in the group pretreated with i.p. injection of compound 6 (yellow bars), RV-I-29 (orange bars) or DK-I-56-1 (green bars) at 1, 3 or 10 mg/kg, as well as topiramate, a known clinically effective anti-migraine drug, at 30 mg/kg (brown bars). The sham group received *i.c.* instillation of the vehicle instead of capsaicin (Sham). Note that only DK-I-56-1, but not Compound 6 or RV-I-29 at 1 mg/kg significantly attenuates capsaicin-induced TCC neuronal activation. Although all the three drugs at 3 and 10 mg/kg significantly attenuates capsaicin-induced TCC neuronal activation, the effectiveness is borderline different at 3 mg/kg (*p* = 0.051) and significantly different at 10 mg/kg (*p* = 0.006) among the three drugs. DK at 10 mg/kg is significantly better than compound 6 (*p* = 0.004) or RV (*p* = 0.009). DK-I-56-1 at 3 mg/kg is better than compound 6 (*p* = 0.014). **(B)** In TG, only DK-I-56-1, but not Compound 6 or RV-I-29, at 1 mg/kg significantly attenuates capsaicin-induced CGRP-ir. The effectiveness is significantly different at 1 mg/kg (*p* = 0.026) among the three drugs. DK at 1 mg/kg is significantly better than compound 6 (*p* = 0.004). **(C)** In dura mater, the three drugs at 1 mg/kg do not significantly suppress capsaicin-induced depletion of dura CGRP-ir. Both Compound 6 and DK-I-56, but not RV-I-29 at 3 mg/kg, significantly attenuates capsaicin-induced CGRP-ir. All of them are effective at 10 mg/kg. The effectiveness is significantly different at 3 mg/kg (*p* = 0.026) among the three drugs. Compound 6 and DK-I-56 are better than RV-I-29 at 3 mg/kg (*p* = 0.011 and 0.043, respectively). Data presentation and statistical analyses are the same as in Figure 3. Differences among groups at the same dosage were compared using the Kruskal–Wallis test followed by *post hoc* Mann-Whitney U test. ^@^ *p* < 0.05 and ^@@^ *p* < 0.01 by Mann-Whitney U test.

We conducted a comparison of the effects of DK-I-56-1, RV-I-29 and Compound 6 on capsaicin-induced neuronal activation, alongside topiramate, an effective anti-migraine drug clinically at the dose of 30 mg/kg, in the TCC (Figure 4A), TG (Figure 4B) and dura mater (Figure 4C). Previously reported results for Compound 6 and topiramate were included for reference (Fan et al., 2018). The sham group and capsaicin-treated vehicle groups remained consistent across all mentioned drug-treated groups. Differences among groups receiving the same dosage were assessed using the Kruskal–Wallis test followed by *post hoc* Mann-Whitney U test. Notably, only DK-I-56-1, at 1 mg/kg, significantly mitigated capsaicin-induced TCC neuronal activation, whereas Compound 6 and RV-I-29 did not demonstrate significant effects at this dosage (Figure 4A). However, at 3 and 10 mg/kg, all three drugs significantly attenuated capsaicin-induced TCC neuronal activation, with differences in efficacy observed particularly at 3 mg/kg (*p* = 0.051) and prominently at 10 mg/kg (*p* = 0.006) among the three drugs. At 10 mg/kg, the efficacy of DK-I-56-1 was significantly superior to that of Compound 6 (*p* = 0.004) or RV-I-29 (*p* = 0.009), and also surpassed that of Compound 6 at 3 mg/kg (*p* = 0.014) (Figure 4A). In the TG, only DK-I-56-1, at 1 mg/kg, exhibited significant attenuation of capsaicin-induced CGRP-ir (*p* = 0.026), with significant differences observed among the three drugs at this dosage. Specifically, the efficacy of DK-I-56-1 at 1 mg/kg was significantly higher than that of Compound 6 (*p* = 0.004) (Figure 4B). In the dura mater, none of the three drugs at 1 mg/kg significantly rescued capsaicin-induced depletion of dural CGRP-ir. However, both Compound 6 and DK-I-56-1, but not RV-I-29, at 3 mg/kg, significantly attenuated this depletion. Notably, significant differences in efficacy were observed at 3 mg/kg among the three drugs (*p* = 0.026), with both Compound 6 and DK-I-56-1 showing higher efficacy than RV-I-29 (*p* = 0.011 and 0.043, respectively). At 10 mg/kg, the efficacies of all three drugs were comparable.

### 3.8 Oral DK-I-56-1 reduced capsaicin-induced TCC neuronal activation

**FIGURE 5:**
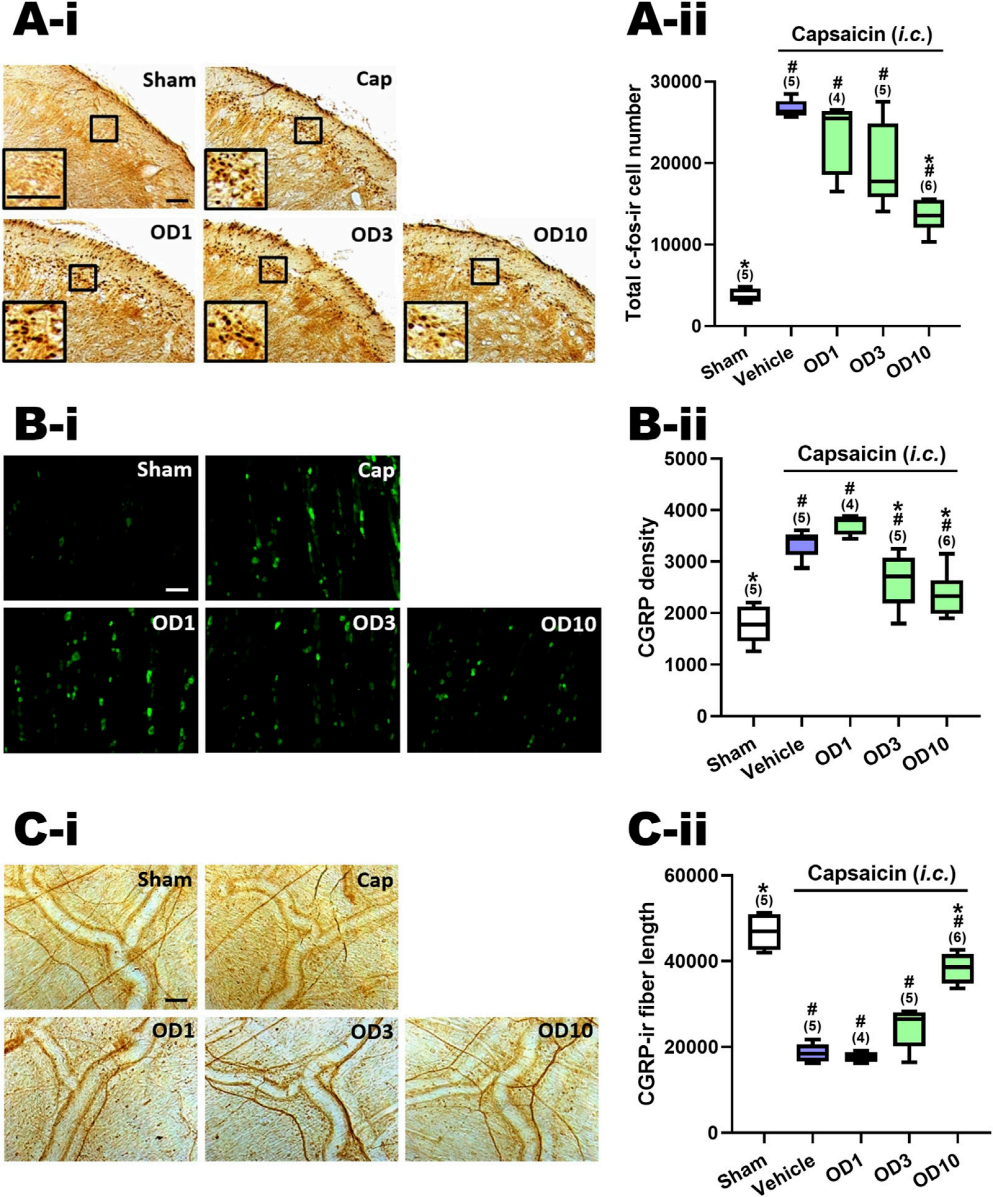
Effects of DK-I-56-1 (*p.o.*) on capsaicin-induced neuronal activation in the trigemino-cervical complex (TCC), trigeminal ganglia (TG) and dura mater. Immunohistograms **(A-i)** and the total number of activated neurons **(A-ii)**, *i.e.* c-Fos-immunoreactive (c-Fos-ir) neurons, in the TCC of rats having received intracisternal (*i.c.*) instillation of 10 nmol capsaicin in the group pretreated with *p.o.* administration of DK-I-56-1 at 1 (OD1), 3 (OD3) or 10 (OD10) mg/kg. The sham group received *ic.* instillation of the vehicle instead of capsaicin (Sham). A close-up image (inset) of c-Fos-containing TCC neurons **(A-i)** is shown in each treatment group. Note that oral treatment of DK-I-56-1 at 10 mg/kg significantly attenuates capsaicin-induced TCC neuronal activation. **(B)** DK-I-56-1 at 3 or 10 mg/kg significantly prevents capsaicin-induced CGRP-ir in TG. **(C-i)** DK-I-56-1 at 10, but not one or 3, mg/kg significantly suppressed capsaicin-induced depletion of dura CGRP-ir. **(C-ii)**. Scale bar, data presentation and statistical analyses are the same as in Figure 2. *p < 0.0167 x i, vs. the Vehicle (Capsaicin) group; ^#^ *p* < 0.0125 x i, *vs.* the Sham group; i: the rank of p value (Supplementary Table S3). (Kruskal–Wallis test followed by *post hoc* Mann-Whitney U test with Benjamin-Hochberg correction). ^@^ *p* < 0.05 by Mann-Whitney U test.

Figure 5A–ii presents a comparison of the total count of c-Fos-ir TCC neurons in groups given varying oral doses of DK-I-56-1 (1–10 mg/kg). Significant differences were observed among the groups (*p* = 0.001 by Kruskal–Wallis test). The Mann-Whitney U test, followed by the Benjamini–Hochberg correction, was applied to compare each treatment group with the capsaicin group (Family 5, Supplementary Table S3) or the sham group (Family 6, Supplementary Table S3). Each family included three or four comparisons in total.

The c-Fos-ir TCC neuronal number exhibited a significant increase in rats subjected to capsaicin administration in comparison with the sham group (*p* = 0.009, Sham vs. Cap, Figure 5A–ii; Supplementary Table S3). Upon oral pretreatment with DK-I-56-1, a significant decrease in capsaicin-induced c-Fos-ir TCC neurons was observed at 10 mg/kg (*p* = 0.006, OD10 vs. Cap, Figure 5A–ii), while doses of one (*p* = 0.221, OD1 vs. Cap, Figure 5A–ii) and 3 (*p* = 0.076, D3 vs. Cap, Figure 5A–ii) mg/kg did not yield significant reductions, relative to the capsaicin group pretreated with vehicle (Figure 5A–ii; Supplementary Table S3). These findings indicate that oral administration of DK-I-56–1 significantly reduces capsaicin-induced neuronal activation in the TCC, although this effect requires a higher dosage compared to *i.p.* DK-I-56-1, which showed suppression at 1 and 3 mg/kg. However, even at 10 mg/kg, oral DK-I-56-1 did not decrease the number of activated TCC neurons to the levels seen in the sham group. (*p* = 0.014, 0.009, and 0.004 for 1 (OD1), 3 (OD3), and 10 (OD10) mg/kg OD *vs.* Sham, respectively; Figure 5A–ii, Supplementary Table S3).

### 3.9 Oral DK-I-56-1attenuated capsaicin-induced CGRP-ir in TG

Figure 5B–ii shows the CGRP-ir in the TG at different oral doses of DK-I-56-1. There were significant differences among groups (*p* = 0.001 by Kruskal–Wallis test). Post hoc analysis using the Mann-Whitney U test with Benjamini–Hochberg correction revealed that following *i.c*. capsaicin instillation, the CGRP-ir in the TG of the capsaicin group was significantly increased compare to that in the sham group (*p* = 0.009, Cap vs. Sham, Figure 5B–ii). Oral administration of DK-I-56-1 significantly suppressed TG CGRP-ir at 3 (*p* = 0.028, OD3 vs. Cap, Figure 5B–ii) and 10 (*p* = 0.011, OD3 vs. Cap, Figure 5C) mg/kg, but not at 1 mg/kg (*p* = 0.05, OD1 vs*.* Cap, Figure 5B–ii), compared to the capsaicin group pretreated with vehicle. However, doses of 3 or 10 mg/kg of DK-I-56-1 failed to reduce TG CGRP-ir levels to those observed in the sham group (*p* = 0.014, 0.028, and 0.045 for 1 (OD1), 3 (OD3) and 10 (OD10) mg/kg OD *vs*. Sham, respectively; Figure 5B–ii, Supplementary Table S3). It is evident that the suppressive efficacy of oral DK-I-56-1 is notably less than that of *i.p.* DK-I-56-1, which completely attenuated CGRP-ir to levels observed in the sham group even at a dose of 1 mg/kg.

### 3.10 Oral DK-I-56-1 reversed capsaicin-induced reduction of CGRP-ir in the dura mater

Figure 5C–ii illustrates the CGRP-ir in the dura mater, quantified by CGRP-ir nerve fiber length, across various treatment groups. Significant differences were observed among these groups (*p* < 0.001 by Kruskal–Wallis test). Post hoc analysis using the Mann-Whitney U test with Benjamini–Hochberg correction revealed that following *i.c.* capsaicin instillation, there was a significant CGRP-ir depletion in the dura mater (Cap vs. Sham, Figure 5C), as evidenced by the CGRP-ir nerve fiber length reduction in the capsaicin group pretreated with vehicle (*p* = 0.009, Cap vs. Sham, Figure 5C–ii) in comparison with the sham group. Oral administration of DK-I-56-1 significantly mitigated capsaicin-induced depletion of CGRP, as indicated by the partial but significant restoration of CGRP-ir fiber length in the dura mater in the groups orally pretreated with DK-I-56-1 at 10 mg/kg (*p* = 0.006, OD10 vs. Cap; *p* = 0.011, OD10 vs. Sham, Figure 5C–ii), but not at one (*p* = 0.462, OD1 vs. Cap, Figure 5C–ii) or 3 mg/kg (*p* = 0.076, OD3 vs. Cap, Figure 5C–ii).

## 4 Discussion

The present study demonstrated that α6GABA_A_R-selective PAMs, DK-1-56-1 (1–10 mg/kg, *i.p.*) and RV-I-29 (3–10 mg/kg, *i.p.*) can ameliorate the central and peripheral responses of TGVS activation in the *i.c.* capsaicin-induced migraine model. Pre-treatment with furosemide, an α6GABA_A_R antagonist, reversed the PAMs’s effect, further support that the anti-migraine effect is possibly mediated via α6GABA_A_R activation. A comparison among DK-1-56-1 and RV-I-29 with their parent compound, Compound 6 as well as topiramate, a clinically used antimigraine-drug, DK-I-56-1 demonstrated better efficacies as a whole. Lastly, we further demonstrated that oral administration of DK-I-56-1 retained the anti-migraine effect in this model.

### 4.1 Deuterated derivatives of compound 6: DK-I-56-1 and RV-I-29

In the 1980s, novel compounds with a PQ backbone were introduced, to display high affinity at the benzodiazepine-site of GABA_A_Rs (α+γ-interface) with pharmacological profiles of either agonists or antagonists (Boast et al., 1985; Czernik et al., 1982). Later, PQ compounds, which bind to a benzodiazepine-insensitive site, the α+β-interface that is distinct from the GABA binding site (α-β+ interface), were identified to be PAMs highly selective to α6GABA_A_Rs PAM (Varagic et al., 2013a; Varagic et al., 2013b; Zhang et al., 1995). Among these PQs, Compound 6 (previously coded as PZ-II-029) with the highest efficacy was selected to elucidate potential clinical indications of α6GABA_A_R-selective PAMs and the role of α6GABA_A_Rs in the pathophysiology of these disorders, such as schizophrenia (Chiou et al., 2018), migraine (Fan et al., 2018) and essential tremor (Huang et al., 2023). For clinical applications, various deuterated derivatives of Compound 6 were synthesized, with retained the α6GABA_A_R-selectivity and better pharmacokinetic profiles, as reported in Knutson et al. (2018). DK-I-56-1 and RV-I-29, previously coded as Compound 8b and 8c, respectively, in Knutson et al. (2018) were selected in this study for further investigation on their pharmacological activities in the migraine model. Among the medicinal chemistry aspects of these deuterated compounds, the significantly prolonged brain bioavailability and plasma half-life (t_1/2_) as compared to the parent Compound 6 are noteworthy. The rat plasma t_1/2_ for Compound 6 (8a), DK-I-56-1 and RV-1-29 were 1.51 ± 0.04 h, 3.54 ± 0.71 h and 2.46 ± 0.15 h, respectively (Knutson et al., 2018).

In the present study, TGVS activation was induced by *i.c.* instillation of capsaicin, an agonist of vanilloid one type of transient receptor potential channels (TRPV1). In small and medium sized unmyelinated TG neurons, TRPV1 are co-expressed with CGRP and substance P (Bae et al., 2004; Caterina and Julius, 2001). TRPV1 activation in TG neurons triggers the release of CGRP that induces vasodilation and neurogenic inflammation within the meninges in experimental animals, mimicking migraine (Meents et al., 2010). Clinically effective anti-migraine drugs, such as valproic acid, topiramate, and a sumatriptan analogue, were shown to suppress both central and peripheral responses in this migraine-mimicking TGVS activation model in rats (Fan et al., 2018; Huang et al., 2018; Cutrer et al., 1996; Cutrer et al., 1999). Here, we found that both deuterated derivatives of Compound 6, DK-I-56-1 and RV-I-29, significantly modulated the TCC neuronal activation, TG CGRP immunoreactivity, and dural CGRP depletion in the capsaicin-induced TGVS activation model, similar to their parent compound, as reported previously (Fan et al., 2018). The efficacy of DK-I-56-1 is relatively superior to Compound 6 and RV-I-29 (Figure 4), which may be attributed to its relatively longer plasma half-life.

### 4.2 Peripheral α6GABA_A_Rs as pharmacological target for migraine and pain

The findings that i.p. furosemide pretreatment significantly blocked the anti-migraine effect of DK-I-56-1 and RV-I-29 further strengthen the basis that these deuterated compounds exhibited the same α6GABA_A_R-selectivity as the parent Compound 6 (Fan et al., 2018). As furosemide is unable to pass through the blood-brain-barrier (BBB) (Seelig et al., 1994), we can exclude the involvement of α6GABA_A_Rs in the CNS. The TG, which is not enclosed by the BBB, is most the likely site-of-action for α6GABA_A_R-selective PAMs in the TGVS.

Hayasaki et al. (2006) reported a functional local GABAergic signalling in the rat TG, where the α6 subunit of GABA_A_Rs (Gabra6) was identified in a subset of TG neurons (Hayasaki et al., 2006). We later also found Gabra6 in TG neurons of rats (Fan et al., 2018), in line with the report from another group (Puri et al., 2012). In mice, we exhibited that Gabra6 was enriched in both TG neurons and the surrounding satellite glial cells (SGCs) (Tzeng et al., 2021). While we were the first group reporting the involvement of TG α6GABA_A_Rs in migraine-related phenotypes, observations from other research teams supported the involvement of TG α6GABA_A_Rs in orofacial-related pain. Administration of DK-I-56-1 repeated for 14 days significantly suppressed trigeminal neuropathic pain in rats (Vasovic et al., 2019). Partial knockdown of the *Gabra6* gene by *Gabra6*-specific siRNA, which reduced Gabra6 expression by 30%, in TG was shown to aggravate the nociceptive responses in rats with trigeminal inflammatory pain (Puri et al., 2012).

### 4.3 Therapeutic potentials of a6GABA_A_R PAMs with enhanced pharmacokinetic profiles

It is noteworthy to point out that α6GABA_A_Rs are non-responsive to benzodiazepines.

Thus, α6GABA_A_R-selective PAMs are unlikely to induce benzodiazepine-like side effects, such as sedation, motor-impairment, and dependence, even though DK-I-56-1 is BBB-permeable (Knutson et al., 2018). In fact, DK-I-56-1 was shown to reverse the ataxic behavior induced by diazepam (Divovic et al., 2022). The motor activity of DK-I-56-1-treated rodents were not suppressed as measured by the open field and rotarod tests (Knutson et al., 2018; Lee et al., 2022). At the dose range of 1–10 mg/kg (*i.p.*), DK-I-56-1 has been shown to alleviate various animal models of centrally-mediated neuropsychiatric disorders, such as essential tremor (Huang et al., 2023), tics disorder (Cadeddu et al., 2021) and schizophrenia. However, repeated administration of DK-I-56-1 at the dose up to 10 mg/kg (*i.p.*) did not cause a dependence-like behaviour in mice, as shown in the conditioned-place preference test (Tzeng et al., 2021). Besides, the development of tolerance in chronic treatment is one of the main drawbacks of benzodiazepines. Interestingly, therapeutic tolerance was not observed in experimental protocol which involve repeated treatments of DK-I-56-1, up to 14 days (Tzeng et al., 2021; Vasovic et al., 2019; Lee et al., 2022).

The enhanced pharmacokinetic profiles due to deuteration combined with our present finding that DK-I-56-1 is orally active, DK-I-56-1 maybe a possible drug candidate to achieve satisfactory therapeutic outcome with a low dosing frequency. Our previous study in nitroglycerin-induced migraine model demonstrated that DK-I-56-1, at the dose of 10 mg/kg (*i.p.*), exerted both prophylactic and abortive effect (Tzeng et al., 2021). It is noteworthy that preliminary toxicological study demonstrated a safe pharmacological profile of PQ compounds, including DK-I-56-1 (Knutson et al., 2018). Moreover, pharmaceutical formulations are being developed to further enhance the stability and bioavailability of DK-I-56-1, *ie*. nanocrystal dispersion (Mitrovic et al., 2023; Mitrovic et al., 2020), which would further enhance the marketability of DK-I-56-1 as treatment for neuropsychiatric disorders, including migraine.

## Data Availability

The raw data supporting the conclusions of this article will be made available by the authors, without undue reservation.

## Abbreviations

α6GABA_A_R, α6 subunit-containing GABA_A_ receptor; aCSF, artificial cerebrospinal fluid; BBB, blood-brain barrier; c-Fos-ir, c-Fos-immunoreactive; CGRP-ir, calcitonin gene-related peptide immunoreactivity; Compound 6, 7-methoxy-2-(4-methoxyphenyl)-2,5-dihydro-3H-pyrazolo[4,3-c]-quinolin-3-one; DK-I-56-1, 7-methoxy-2-(4-methoxy-d3-phenyl)-2,5-dihydro-3H-pyrazolo-[; 4,3-c]quinolin-3-one; GABA_A_R, GABA_A_ receptor; *i.c.*, intra-cisternal injection; *i.p.*, intraperitoneal injection; *p.o.*, oral administration; PAM, positive allosteric modulator; TCC, trigeminal cervical complex; TG, trigeminal ganglia; TGVS, trigeminovascular system; TMJ, temporomandibular joint; TNC, trigeminal nucleus caudalis; TRPV1, transient receptor potential vanilloid type-1 channel. RV-I-29, 7-(methoxy-d3)-2-(4-methoxyphenyl)-2,5-dihydro-3H-pyrazolo[4,3-c]quinolin-3-one.
