# Supplementary material for: The deuterated pyrazoloquinolinone targeting α6 subunit-containing GABAA receptor as novel candidate for inhibition of trigeminovascular system activation: implication for migraine therapy
Source: Front Pharmacol. 2024 Aug 26;15:1451634. doi: 10.3389/fphar.2024.1451634 (PMC11381949; doi:10.3389/fphar.2024.1451634)
Supplement: Supplementary file 1 [file Table1.DOCX]

**Table S1.** Statistical parameters derived from the Kruskal-Wallis test followed by the Mann-Whitney U test with Benjamini-Hochberg correction for paired comparisons with the vehicle-pretreated capsaicin group and sham group.

| Family-1^a^ | TCC | | TG | | Dura | |
| --- | --- | --- | --- | --- | --- | --- |
|  | P value  (rank)^b^ | BHC value^c^ | P value  (rank)^b^ | BHC value^c^ | P value  (rank)^b^ | BHC value^c^ |
| Cap *vs*. D1 | 0.025* (3) | 0.0375 | 0.004* (1) | 0.0125 | 0.337  (4) | 0.05 |
| Cap *vs*. D3 | 0.011* (2) | 0.025 | 0.011* (3) | 0.0375 | 0.019* (2) | 0.025 |
| Cap *vs*. D10 | 0.006* (1) | 0.0125 | 0.006* (2) | 0.025 | 0.006* (1) | 0.0125 |
| Cap *vs*. D3+Furo | 0.522 (4) | 0.05 | 0.136  (4) | 0.05 | 0.088  (3) | 0.0375 |
| Family-2^d^ |  |  |  |  |  |  |
| Sham vs. Cap | 0.004*  (1) | 0.01 | 0.006*  (1) | 0.01 | 0.004*  (1) | 0.01 |
| Sham *vs*. D1 | 0.004* (1) | 0.01 | 0.068 (3) | 0.03 | 0.004*  (1) | 0.01 |
| Sham *vs*. D3 | 0.011* (4) | 0.04 | 0.221 (4) | 0.04 | 0.011* (4) | 0.04 |
| Sham *vs*. D10 | 0.006* (3) | 0.03 | 0.754 (5) | 0.05 | 0.006* (3) | 0.03 |
| Sham *vs*. D3+Furo | 0.011* (4) | 0.04 | 0.027  (2) | 0.02 | 0.011*  (4) | 0.04 |

Thirty minutes before capsaicin *i.c.* instillation, rats were pretreated with DK at 1 (D1), 3 (D3) and 10 (D10) mg/kg, respectively, or with 3 mg/kg DK plus furosemide (D3+Furo).

^a^Each pretreatment group was compared with the vehicle-pretreated group (Cap).

^b^The rank of the P value of the comparing pair in the comparison family, Family-1 and Family-2, respectively.

^c^Benjamini-Hochberg critical value: (i/m)Q, where i is the rank of the P value of the comparing pair, and m is total number (4 in Family-1 and 5 in Family-2) of comparisons, Q: 0.05.

^d^Each pretreatment group was compared with the sham group (Sham).

**Table S2.** Statistical parameters derived from the Kruskal-Wallis test followed by the Mann-Whitney U test with Benjamini-Hochberg correction for paired comparisons with the vehicle-pretreated capsaicin group and sham group.

| Family-3^a^ | TCC | | TG | | Dura | |
| --- | --- | --- | --- | --- | --- | --- |
|  | P value  (rank)^b^ | BHC value^c^ | P value  (rank)^b^ | BHC value^c^ | P value  (rank)^b^ | BHC value^c^ |
| Cap *vs*. R1 | 1.0  (4) | 0.05 | 0.144 (4) | 0.05 | 0.855  (4) | 0.05 |
| Cap *vs*. R3 | 0.006* (1) | 0.0125 | 0.011* (2) | 0.025 | 0.286 (3) | 0.0375 |
| Cap *vs*. R10 | 0.006* (1) | 0.0125 | 0.006* (1) | 0.0125 | 0.006* (1) | 0.0125 |
| Cap *vs*. R3+Furo | 0.011* (3) | 0.0375 | 0.011*  (2) | 0.025 | 0.273  (2) | 0.025 |
| Family-4^d^ |  |  |  |  |  |  |
| Sham vs. Cap | 0.004*  (1) | 0.01 | 0.006*  (1) | 0.01 | 0.004*  (1) | 0.01 |
| Sham *vs*. R1 | 0.006* (2) | 0.02 | 0.047 (3) | 0.03 | 0.006*  (2) | 0.02 |
| Sham *vs*. R3 | 0.006* (2) | 0.02 | 0.221 (4) | 0.04 | 0.011* (5) | 0.05 |
| Sham *vs*. R10 | 0.006* (2) | 0.02 | 0.602 (5) | 0.05 | 0.006* (2) | 0.02 |
| Sham *vs*. R3+Furo | 0.006* (2) | 0.02 | 0.028  (2) | 0.02 | 0.006*  (2) | 0.02 |

Thirty minutes before capsaicin *i.c.* instillation, rats were pretreated with RV at 1 (R1), 3 (R3) and 10 (R10) mg/kg, respectively, or with 3 mg/kg RV plus furosemide (R3+Furo).

^a^Each pretreatment group was compared with the vehicle-pretreated group (Cap).

^b^The rank of the P value of the comparing pair in the comparison family, Family-3 and Family-4, respectively.

^c^Benjamini-Hochberg critical value: (i/m)Q, where i is the rank of the P value of the comparing pair, and m is total number (4 in Family-3 and 5 in Family-4) of comparisons, Q: 0.05.

^d^Each pretreatment group was compared with the sham group (Sham).

**Table S3.** Statistical parameters derived from the Kruskal-Wallis test followed by the Mann-Whitney U test with Benjamini-Hochberg correction for paired comparisons with the vehicle-pretreated capsaicin group and sham group.

| Family-5^a^ | TCC | | TG | | Dura | |
| --- | --- | --- | --- | --- | --- | --- |
|  | P value  (rank)^b^ | BHC value^c^ | P value  (rank)^b^ | BHC value^c^ | P value  (rank)^b^ | BHC value^c^ |
| Cap *vs*. OD1 | 0.221  (3) | 0.05 | 0.05*  (3) | 0.05 | 0.462  (3) | 0.05 |
| Cap *vs*. OD3 | 0.076 (2) | 0.033 | 0.028* (2) | 0.033 | 0.076 (2) | 0.033 |
| Cap *vs*. OD10 | 0.006* (1) | 0.0167 | 0.011* (1) | 0.0167 | 0.006* (1) | 0.0167 |
| Family-6^d^ |  |  |  |  |  |  |
| Sham vs. Cap | 0.009*  (2) | 0.025 | 0.009*  (1) | 0.0125 | 0.009*  (1) | 0.0125 |
| Sham *vs*. OD1 | 0.014* (4) | 0.05 | 0.014* (2) | 0.025 | 0.014*  (4) | 0.05 |
| Sham *vs*. OD3 | 0.009* (2) | 0.025 | 0.028* (3) | 0.0375 | 0.009* (1) | 0.0125 |
| Sham *vs*. OD10 | 0.004* (1) | 0.0125 | 0.045* (4) | 0.05 | 0.011* (3) | 0.0375 |

Thirty minutes before capsaicin *i.c.* instillation, rats were pretreated with DK orally at 1 (OD1), 3 (OD3) and 10 (OD10) mg/kg, respectively.

^a^Each pretreatment group was compared with the vehicle-pretreated group (Cap).

^b^The rank of the P value of the comparing pair in the comparison family, Family-5 and Family-6, respectively.

^c^Benjamini-Hochberg critical value: (i/m)Q, where i is the rank of the P value of the comparing pair, and m is total number (3 in Family-5 and 4 in Family-6) of comparisons, Q: 0.05.

^d^Each pretreatment group was compared with the sham group (Sham).
